# Impact of maternal breast cancer on school-aged children in Saudi Arabia

**DOI:** 10.1186/1756-0500-7-261

**Published:** 2014-04-23

**Authors:** Faten Al-Zaben, Samia M Al-Amoudi, Basem Salama El-deek, Harold G Koenig

**Affiliations:** 1 Faculty of Medicine, King Abdulaziz University, Jeddah, Saudi Arabia; 2 Sheikh Mohammed Hussein Al-Amoudi Breast Cancer Center of Excellence and Faculty of Medicine, King Abdulaziz University, Jeddah, Saudia Arabia; 3 Community Medicine, Faculty of Medicine, King Abdulaziz University, Jeddah, Saudi Arabia; 4 Psychiatry & Behavioral Sciences and Center for Spirituality, Theology and Health, Duke University Medical Center, Durham, NC 27705, USA

**Keywords:** Breast, Cancer, Children, Psychology, Chronic illness, Psychosocial, Sociology, Saudi, School-aged, Mothers

## Abstract

**Background:**

We examine whether mothers with breast cancer told their children about the diagnosis, explore mothers’ perceptions of the impact of doing so on the mother-child relationship, and assess perceptions of how this affected the children.

**Methods:**

A convenience sample of 28 women with breast cancer ages 35 to 60 was interviewed using a 39-item close-ended questionnaire at the Al-Amoudi Breast Cancer Center of Excellence, King Abdulaziz University, Jeddah, Saudi Arabia. Inclusion criteria were having a diagnosis of breast cancer and having school-aged children (ages 5 to 16 years). Questions were asked concerning each child (n = 99).

**Results:**

The majority of women (75%) told their children about the diagnosis, and explained the treatment (61%). In most cases, telling the children had a positive effect on how the children treated their mothers (84%), on the maternal-child relationship (80%), and on the personality and behavior of the child (90%). The most common negative reaction by children was increased clinging behavior to the mother (15%). Despite the perceived positive impact on the mother-child relationship and on the child’s overall behavior towards the mother, school performance suffered as a result (77%).

**Conclusions:**

These preliminary results suggest that when a mother with breast cancer tells a child about the diagnosis and discusses it with them, this often results in an improvement in the maternal-child relationship. However, the knowing the mother’s diagnosis may adversely affect the child’s school performance, which will need to be anticipated and addressed with formal counseling if it persists.

## Background

Breast cancer is a major health problem in the Kingdom of Saudi Arabia (KSA) [1], and is the most common cancer in women, representing 26% of all newly diagnosed cancers in this gender [2]. Late stage at presentation in young women is a serious problem [3]. A young age at diagnosis means that more women are likely to be diagnosed while having children of school age living at home. Therefore, women need skills to communicate this diagnosis to their children.

Breast cancer affects not only the woman but also the entire family. Research has only just begun to explore these sensitive issues [4,5]. Little attention, however, has been paid to whether, what, and how children should be told about their mother’s diagnosis. In conservative communities like those in Saudi Arabia, cancer is still a sensitive issue to talk about or discuss openly with children and the decision is usually left to judgment of the parents (who often have little guidance on how to proceed).

Studies have found that mothers mainly rely on their children to ask questions and tend not to volunteer information, sometimes misinterpreting their children’s reactions to the illness [6]. Many children report feeling inadequately prepared to see their mother suffer, and those as young as seven years of age are often more aware of the life-threatening nature of cancer than parents realize [7]. The main sources of information that children have about breast cancer are either relatives or the media, so the information they receive is often neither adequate or accurate.

### Objectives

To our knowledge, no studies have yet systematically examined the impact of the diagnosis of breast cancer on the school-aged children of women so affected in KSA or the Middle East, where religious and cultural factors emphasize the importance of family relationships. The present study seeks to explore the impact of a breast cancer diagnosis on children’s psychosocial stability and mother-child relationship as perceived by their mothers. We examine [1] whether mothers with breast cancer told their children about their diagnosis, [2] explore mothers’ perceptions of the impact of doing so on the mother-child relationship, and [3] assess mothers’ perception of how this affected their children’s psychosocial stability and school performance.

## Methods

In this descriptive study, women diagnosed with breast cancer and involved in the Sheikh Mohammed Hussein Al-Amoudi Breast Cancer Center of Excellence were approached over a one-month period to participate in the study. Inclusion criteria were [1] diagnosis of breast cancer (any time, but preferably within the past 12 months), [2] stage I-III breast cancer (stage IV excluded), and [3] having children ages 5 to 16 attending school (children in full time special education excluded).

Women meeting the inclusion criteria were contacted by telephone to set up an appointment at the Center. A single trained research assistant conducted all interviews face-to-face. The study was explained to participants and their oral consent was obtained. The ethics committee of King Abdulaziz University Hospital approved the study.

The research assistant asked a series of 39 closed-ended questions to women who participated in the study. Each interview took about 45–60 minutes to complete since women often wanted to talk about their answers. Assessed were demographic characteristics, attitudes of mothers toward informing their children of the diagnosis and discussing it with them, the impact that mothers perceived the diagnosis had on the children’s behavior toward them, and the effect that this had on the children’s school performance. These questions were developed and refined by faculty at the King Abdulaziz University’s Breast Cancer Center of Excellence. In this exploratory study, close-ended questions were chosen in order to determine the proportion of mothers with breast cancer who told their children about the diagnosis, to identify which perceptions of the impact of disclosure on the children were most prevalent, and detect the percentage of mothers who thought their children’s psychosocial stability and school performance improved or deteriorated as a result of doing so.

## Results

A total of 28 women with breast cancer were interviewed about the impact of the diagnosis on their school-age children (n = 99). The average age of participants was 48.9 years (range 35 to 60), about two-thirds were Saudi nationals, and 57% had at least a high school education (Table 1). The majority of women were currently married (61%) and lived with their spouse and children (64%). The average number of children was four, with a range of 1 to 10, and 56% were female. The average age of the children when mothers were diagnosed with breast cancer was 10.7 years.

**Table 1:** Demographic characteristics (n = 28)

	**Mean (SD) (range)**	**% (n)**
Age, y	48.9 (7.1) (35–60)	--
Nationality, % Saudi (n)	--	67.9 (19)
Years in country (if not Saudi), y	26.3 (11.5) (9–48)	--
Education, % high school graduate (n)	--	57.1 (16)
Marital status, % (n)		
Married	--	60.7 (17)
Widowed	--	21.4 [6]
Divorced	--	21.4 [6]
Living situation, % (n)		
With children only	--	28.6 [8]
Spouse and children	--	64.3 (18)
Spouse, children, & relatives	--	7.1 [2]
No. of children	4.0 (2.0) (1–10)	--
Age of children at diagnosis, y	10.7 (3.0) (1–24)	--
Gender, % female (n)	--	55.5% (55)

### Informing children of the diagnosis

Three-quarters (75%) of women informed their children of the diagnosis (Table 2). The average age of the first child told was 13.0 years, of the second child told was 8.7 years, and of the third child told was 5.9 years. Of mothers who told their children about the diagnosis (n = 21), the three most common reasons for doing so were that the child would [1] hear it from them, [2] be more scared if not told, and [3] be empowered by knowing. Less common reasons were so the children would cope better with the disease and that it would increase their faith in God’s reward.

**Table 2:** Attitude of mother towards informing children of diagnosis (n = 28)

	**Mean (SD)**	**% (n)**
Told children diagnosis, % yes	--	75% (21)
Age of child when told (ages 5–16), y		
1st child	13.0 (2.5)	--
2nd child	8.7 (1.9)	--
3rd child	5.9 (1.8)	--
Why told (n = 21), % yes (n)		
Know from me	--	47.6 (10)
Child more scared if not told	--	42.8 [9]
Empower them	--	42.8 [9]
Help cope better with disease	--	33.3 [7]
Increase faith in God’s reward	--	23.8 [5]
Why wait or not tell, % yes (n)		
No need to scare	--	35.7 (10)
Issue for adults only	---	25.0 [7]
No benefit	--	10.7 [3]
Might damage personality	--	10.7 [3]
Don’t want to hurt child	--	0.0 (0)
Talked to child about cancer, % yes (n)	--	71.4 (20)
What told child, % yes (n)		
A disease like any disease	--	39.3 (11)
New treatment with good results	--	39.3 (11)
Allah’s love to test us and reward	--	39.3 (11)
Will take time to recover	--	25.0 [7]
Believe Allah will be there and pray	--	21.4 [6]
Explained treatment to child, % yes (n)	--	60.7 (17)
What told child about treatment, % yes (n)		
Treatment will take long time	--	57.1 (16)
Might lose hair	--	50.0 (14)
Might need surgery	--	39.3 (11)
Will suffer from side effects	--	25.0 [7]
Won’t be able to do things like before	--	14.3 [4]
Love them and need their help	--	14.3 [4]

When mothers were asked why they might wait or not tell their children the diagnosis, about one-third (36%) said they didn’t want to scare the child and one-quarter (25%) said that this was an issue for adults only. Only a few women indicated that there was no benefit to telling or that knowing the diagnosis might injure the child’s personality.

Mothers were also asked if they sat down with and talked to their child about the cancer (vs. simply informing them of the diagnosis). Again, most of the women who told their children (20 out of 21) took the time to discuss the diagnosis and let their children ask questions. With regard to what they told their children, the most common themes were that [1] this disease was just like any other disease (39%); [2] there were new treatments for breast cancer with good results (39%); and [3] the disease was an expression of God’s love as a test that would result in a reward (39%). About a quarter indicated that it would take time to recover, but that God would be there to help and so encouraged them to pray.

The majority of women (61%) also explained the treatment to their children. The most common explanation was that the treatment would take a long time (57%) and that they might lose their hair (50%). Less frequent was that they might need surgery (39%) or suffer with side-effects (25%). Only a few mothers indicated that they wouldn’t be able to do things like they did before and that they loved their children and needed their help.

### Impact on child

Interestingly, most responses by mothers indicated that telling their children (n = 99) about their breast cancer diagnosis and discussing it with them actually had a positive impact (Table 3). With regard to the way their children treated them, in 84% of cases mothers indicated that there was a positive change (i.e., a change for the better). In only two cases did mothers say the change was negative, one indicating that the child became angry and the other that the child withdrew from her. With regard to changes in the child’s personality, in 90% of cases the change was reported to be positive and in only 1% (1 of 99 children) was it negative, i.e., the child became more emotional.

**Table 3:** Impact of diagnosis on children’s behavior and performance (n = 99)

	**% (n)**
Child changed way treat mother, % yes	
Positive change, % yes	83.8 (83)
Negative change, % yes (of all children)	2.0 [2]
No change	14.1 (14)
How change if negative, % yes	
Angry with mother	1.0 [1]
Became withdrawn	1.0 [1]
Hate mother	0.0 (0)
Don’t know	0.0 (0)
Change in child’s personality, % yes	
Positive personality change	89.9 (89)
Negative change	1.0 [1]
No change	9.1 [9]
How change if negative, % yes	
More emotional	1.0 [1]
Became withdrawn	1.0 [1]
Very scared	0.0 (0)
Withdrawn	0.0 (0)
Other	0.0 (0)
Reactions of child, % yes	
Cling to mother	15.2 (15)
Resent needed to help	13.1 (13)
Afraid might get cancer also	11.1 (11)
Behaved badly to cover	7.1 [7]
Got sick to get attention	6.1 [6]
Withdraw from mother	2.0 [2]
Relationship affected by diagnosis, % yes (n)	
Positive change	79.8 (79)
Negative change	1.0 [1]
No change	19.2 (19)
School performance affected, % yes (n)	
Positive change	8.1 [8]
Negative change	76.8 (76)
No change	15.2 (15)

With regard to immediate reactions from children after being told, the most common response was increased clinging to the mother (15%) and the second most common response, surprisingly, was that they resented needing to help (13%). Less common reactions were that children became afraid that they might get cancer also (11%), began behaving badly to cover their feelings (7%), acted sick to get attention (6%), or withdrew from the mother (2%). Overall, though, the result of telling the diagnosis to the child was that the relationship between mother and child changed in a positive direction (80% of cases), whereas in only 1 of 99 children did it worsen the relationship.

Although in the vast majority of cases there was improvement in the way the child treated the mother, in the personality of the child, and in the relationship between mother and child, school performance did appear to suffer. In over three-quarters (77%), school performance worsened, whereas in only 8% did it improve.

## Discussion

To our knowledge, this is the first systematic report from Saudi Arabia and the Middle East on what happens when women with breast cancer tell their school-aged children about their diagnosis. Given the importance of family in this part of the world and the frequency of breast cancer, it is surprising that researchers have not examined this topic previously. There has been research on and discussions about this issue in Western countries [6,8], although there is concern that such results cannot be applied to Arabic or predominantly Muslim areas of the world where cultural and religious factors have such a strong influence.

The present research uncovered several important findings. First is that the vast majority of women with breast cancer told their children of the diagnosis (75%) and discussed the diagnosis and its treatment with them (71%). There has been a trend in this part of the world not to give bad news (such as a diagnosis of cancer) to patients, nor to have such honest discussions with family members, particularly young children, about the diagnosis. Perhaps the close relationship between mothers and their children encourages open explanations and discussions.

The second most important finding was that discussing the diagnosis with the child actually improved the way the child treated their mother (84%), improved the way the child acted (personality), and improved the overall relationship with the mother (80%). All of these are encouraging and should help reassure women with breast cancer that telling their children is the right thing to do in most cases.

The third most interesting finding was that, while the relationship with the mother improved, school performance suffered for most children (77%). Knowing about their mother’s diagnosis of cancer, then, did affect children in perhaps a less conscious way that revealed itself in poorer academic performance. Given the importance of family and relationships between family members, attention may have been diverted away from less important issues (school) and redirected toward the relationship with the mother.

Other studies from Western countries have documented what happens when a mother with breast cancer tells her children about the diagnosis. Those studies have found that such disclosure often affects the well-being of children, as well as their relationships with peers [6]. Children confronted with serious illness in a parent may in some cases experience traumatic stress responses and even post-traumatic stress disorder (PTSD) [5]. Children’s reactions may be related to how their parent is coping, since evidence suggests that increased maternal depression in breast cancer may lead to psychological problems in children. However, the existing evidence on children’s reactions is often contradictory, with both high rates of psychological disturbance and good functioning reported [8].

### Limitations

The sample was one of convenience, was relatively small, and was acquired from one university setting in Jeddah, Saudi Arabia, which may limit the ability to generalize the findings to other women with breast cancer in rural areas or those seeking care in non-university settings. We did not collect information about subjects who refused to participate in the study. Thus, women who were more afraid or uncomfortable about the study’s objectives may have been under-represented in the study. It would also have been interesting to interview the children directly to determine from them how they perceived being told about their mother’s breast cancer, although this was not possible within the structure of the current study. Finally, although we described the impact of learning about the diagnosis for 99 children, these were not independent events since the perceived responses came from 28 women who on average each reported the responses of 4 children. This too may have affected the findings in this study.

### Strengths

This is the first report from a Middle Eastern country on whether women with breast cancer told their children about the diagnosis, why they did so, and how women perceived their children were affected. Cultural and religious factors have an enormous influence on the perceptions and behaviors of women with breast cancer, and findings from Western countries cannot be applied to women from this part of the world. Thus, even these preliminary findings will be of value as a snapshot of how women with breast cancer in Islamic countries deal with their children when diagnosed with this disease.

## Conclusions

The cancer experience (with the diagnosis, threat of recurrence, and recurrence) is a stressful process that influences a woman’s closest relationships, especially with those who most depend on her. Mothers may wish to protect their children from the stress of knowing that they have cancer by concealing this diagnosis from them. In the present study, however, the majority of women with breast cancer told their school-aged children about the diagnosis and discussed it with them. In most cases, telling their child had a positive effect on the maternal-child relationship and on the child’s behavior, as perceived by mothers. The only exception was that children’s school performance appeared to suffer, which may have been due to a re-direction of their attention to what their mothers were going through. Although further research in more representative samples is clearly warranted to confirm the results of this study, the present findings have at least preliminary implications.

First, women with breast cancer should be encouraged to tell their children of the diagnosis, discuss it with them, and allow their children to ask questions. They can be reassured that in the vast majority of cases, doing so will likely have a positive impact on the relationship with their children. Second, women should be aware that their children’s school performance may suffer as a result of knowing about the diagnosis and dealing with the emotional issues related to that knowledge. If this occurs and does not diminish over time, then women should be encouraged to seek counseling for their children to help them adjust to their mother’s diagnosis and the implications thereof.

## Competing interests

The authors declare that they have no competing interests, neither financial nor non-financial.

## Author’s contributions

SMAA, FAZ, BSE, and HGK wrote the proposal and designed the study. SMAA and FAZ collected the data. SMAA, HGK, and FAZ drafted the final report, with BSE contributing. BSE analyzed the data, with input from SMAA and HGK. All authors read and approved the final manuscript.

## References

[B1] Ibrahim EM Zeeneldin AA Sadiq BB Ezzat AA The present and the future of breast cancer burden in the Kingdom of Saudi Arabia Med Oncol 2008 25 387 393 10.1007/s12032-008-9051-5 18317955

[B2] Dandash KF Al-Mohaimeed A Knowledge, attitudes, and practices surrounding breast cancer and screening in female teachers of Buraidah, Saudi Arabia Int J Health Sci (Qassim) 2007 1 61 71 21475453 PMC3068667

[B3] El Saghir NS Khalil MK Eid T El King AR Charafeddin M Geara F Trends in epidemiology and management of breast cancer in developing Arab countries: a Literature and registry analysis Int J Surg 2007 5 225 233 10.1016/j.ijsu.2006.06.015 17660128

[B4] Osborn T The psychosocial impact of parental cancer on children and adolescents: a systematic review Psychooncology 2007 16 101 126 10.1002/pon.1113 17273987

[B5] Edwards L Watson M St James-Roberts I Ashley S Tilney C Brougham B Osborn T Baldus C Romer G Adolescent’s stress responses and psychological functioning when a parent has early breast cancer Psychooncology 2008 17 1039 1047 10.1002/pon.1323 18318453

[B6] Vannatta K Grollman JA Noll RB Gerhardt CA Impact of maternal breast cancer on the peer interactions of children at school Psychooncology 2008 17 252 259 10.1002/pon.1232 17575564

[B7] Forrest G Plumb C Ziebland S Stein A Breast cancer in young families: a qualitative interview study of fathers and their role and communication with their children following the diagnosis of maternal breast cancer Psychooncology 2009 18 96 103 10.1002/pon.1387 18618898

[B8] Watson M St James-Roberts I Ashley S Tilney C Brougham B Edwards L Baldus C Rober G Factors associated with emotional and behavioural problems among school age children of breast cancer patients Br J Cancer 2006 94 43 50 10.1038/sj.bjc.6602887 16317432 PMC2361079

